# The evolving landscape of airway education in anesthesia for head and neck surgery

**DOI:** 10.1097/ACO.0000000000001572

**Published:** 2025-09-29

**Authors:** Alexander Fuchs, Vladimir Nekhendzy, Robert Greif

**Affiliations:** a Department of Anaesthesiology and Pain Medicine, Inselspital, Bern University Hospital, University of Bern, Bern, Switzerland; b Department of Anesthesiology, Perioperative and Pain Medicine, Stanford University Medical Center, Stanford, California, USA; c Faculty of Medicine, University of Bern, Bern, Switzerland

**Keywords:** difficult airway, ear nose and throat anesthesia, education, head and neck anesthesia, head and neck surgery, teaching

## Abstract

**Purpose of review:**

Airway management for head and neck surgery presents some of the most unique and complex challenges for both adult and pediatric patient populations. High level of technical proficiency is essential, and the technical skill set should be complemented by a variety of solid nontechnical skills to ensure adequate interdisciplinary and interprofessional collaboration. Little is known about state-of-the-art teaching methods addressing these important skills.

**Recent findings:**

This article explores recent advancements in airway education, focusing on how technical and nontechnical skills could be best integrated into head and neck anesthesia teaching curricula with the goals of optimizing airway management outcomes and patient safety.

**Summary:**

We examined the role of simulation-based training, competency-based medical education, and the emphasis on human factors in head and neck anesthesia to facilitate shaping modern curricula.

**KEY POINTS**

- Each head and neck anesthesia unit is encouraged to establish a formal airway management curriculum.
- Besides the theoretical knowledge, simulation on a part-task trainer to learn technical competences, and full-scale simulation to gain proficiency in team competences, seems ideal before taking care of head and neck patients.
- Follow-up on new developments in educational approaches, such as extended reality, video learning, escape rooms, and innovations in simulation and debriefing.
- Establish short interactive teaching units distributed over time for training and rehearsal of (rare) airway management situations.

**OBJECTIVE**

- Briefly introduce the shift from traditional apprenticeship models to more structured, competency-based approaches that explicitly address both technical and nontechnical skills.
- Analyze existing teaching methods for both skill sets in this specific field.
- Identify training gaps and suggest improvements.

## INTRODUCTION

Airway management for head and neck surgery presents a unique set of challenges due to the complexities of the upper and lower airway anatomy, the proximity of major vascular structures, and the shared airway considerations [1]. There is a high risk of airway-related perioperative complications [2], which can jeopardize patients’ outcomes [3]. These complexities demand a high level of anesthesiologist’s proficiency with both technical and nontechnical skills [4]. However, limited structured and focused airway training for head and neck surgical patients exists in the majority of anesthesia training programs [5,6].

Competent and safe airway management in head and neck surgery requires the anesthesiologist to be adept with a wide variety of devices and techniques, such as video laryngoscopy, flexible intubation scope, supraglottic airway ventilation, front-of-neck access, tubeless ventilatory techniques (high-flow nasal oxygen and jet ventilation), as well as safe extubation strategies. Furthermore, clinicians must be prepared for specific emergencies, including sudden loss of the airway, bleeding, and venous air embolism.

Equally important is the teaching of nontechnical skills [7]. Effective communication with the surgical team, joint planning, and real-time decision making are crucial in this interdisciplinary setting. Teaching interventions must foster collaborative behaviors to prevent miscommunication and avoidable complications.

This review outlines contemporary evidence-based teaching strategies for technical and nontechnical skills in anesthesia for head and neck surgery. Few articles have been published on the teaching of airway management in head and neck anesthesia. We aimed to highlight recent trends, provide practical guidance, and support the development of structured training pathways for anesthesiologists; however, most of the educational evidence is very indirect for such a high-stakes field. New educational approaches – spaced learning [8], mastery of skills [9] and deliberate practice [10], simulation-based medical education [11,12], in situ training with video-assisted debriefing [13], self-directed video learning [14^■^,15^■^], cognitive aids [16] and checklists [17], and immersive technologies such as augmented and virtual reality (VR) [18,19] – have the potential to improve operator’s performance, promote patient safety, and enhance learner engagement.

## THE TEACHING OF TECHNICAL SKILLS IN ANESTHESIA FOR HEAD AND NECK SURGERY

### Core competency

Safe airway management in adults and children is paramount, given varying degrees of airway compromise, frequent difficulties with visualization and tracheal access (swelling, postradiation tissue fixation and restriction, bleeding, etc.), and challenging shared airway considerations with the surgeons (Table 1).

**Table 1.** Competences for a competency-based curriculum for head and neck anesthesia [20]

Medical knowledge and patient care during the perioperative period	
Preoperative assessment	Patient assessment and history. Physical exam and standard airway assessment tests. Advanced airway assessment including preoperative imaging (e.g. computed tomography scan) and nasal endoscopy
	Understand unique anatomical and physiological challenges presented by head and neck pathology (e.g. risks of airway obstruction, shared airway, and intraoperative bleeding)
	Develop a comprehensive, individualized patient-centered airway management and anesthetic plan based on the patient’s assessment and the surgical procedure
Intraoperative management	Mastery of various basic and advanced airway management techniques. Clinical application of a difficult airway management algorithm adopted in the institution. Perioxygenation and apneic oxygenation strategies, video laryngoscopy, flexible scope intubation, combined techniques, awake intubation, rescue techniques and emergency front of the neck access, safe and staged extubation strategies, tracheostomy, and laryngectomy care management
	Demonstrate expertise in clinical pharmacology of inhaled and intravenous anesthetics, local anesthetics used for airway topicalization, and in fluid and blood management
	Recognition and management of surgical-specific requirements for proper intraoperative patient positioning. Demonstrate management of the shared airway, and head and neck surgical complications, such as intraoperative hemorrhage, venous air embolism, and surgical emergencies (e.g. posttonsillectomy bleeding, tracheostomy problems)
	Become familiar with anesthetic and airway considerations for a safe and effective use of specialized surgical and anesthetic equipment (e.g. lasers, nerve monitors, high-frequency jet ventilators, high-flow nasal oxygen)
Emergence and postoperative care	Promote safe and controlled emergence from anesthesia, Focus on safe extubation (staged extubation, if required) and adequate, individualized postoperative airway management
	Management of postoperative oxygenation, ventilation, hemodynamics, pain, nausea, and nausea/vomiting
	Identification and prompt treatment of immediate postoperative complications, such as hypoxia, hemodynamic instability (e.g. hematoma formation, bleeding), airway obstruction, and compromised ventilation
Team communication and collaboration	
Effective team communication	Clear, concise, and timely communication with the surgical team, nurses, and other specialists, especially during critical moments. Situation awareness, concise decision making, close-loop communication
Teamwork	Task distribution and management. Collaborative leadership, supporting all team members as an integral part of the care team. Anticipation of the different roles for team members managing crisis (e.g. shared airway, intraoperative bleeding)
Patient and family interaction	Explaining anesthetic risks and care plan to patients and their families. Promotion of shared decision-making. Ensuring adequate patient’s informed consent
Professionalism and systems-based practice	
Ethical behavior	Adherence to professional codes of conduct. Maintaining patient confidentiality. Demonstrating compassion and empathy
Quality and safety	Commitment to patient safety and quality improvement initiatives. Adherence to established protocols, algorithms and checklists. Analysis of critical incidents and root causes
Leadership and advocacy	Ability to lead a team and provide a collaborative, task-oriented approach during standard and emergency situations. Advocate for optimal patient care at all times
Well-being of healthcare professionals	Caring for the entire team in the operating room to ensure patients’ optimal and quality treatment as a first priority. Debriefing as necessary, with special attention to the psychological and physical well-being of team members

### Challenges and specific requirements for the key technical skills of advanced airway management

#### Video laryngoscopy

Training in both standard and hyperangulated video laryngoscope blades, as well as the use of bougies and stylets, are essential [21–23]. Video laryngoscopy with standard blades can also teach direct laryngoscopy and may be viewed as a preferred teaching tool [24^■■^,25^■^].

#### Flexible scope intubation

Both awake and asleep flexible scope intubations have relatively high failure rates in patients with head and neck pathology, ranging from 9 to 60%. The most common reasons for flexible scope intubation failure in head and neck patients include inability to identify the glottis, difficulties with tracheal access, and a lack of confidence, skills, judgment, and equipment [26,27]. In recent years, the combination of video laryngoscopy and a flexible intubation scope has been introduced, showing higher first attempt success rates compared to video laryngoscopy alone in patients with anticipated difficult airway [26,28]. The use of this approach requires anesthesiologists to be proficient in both techniques before combining them.

#### Awake tracheal intubation

Proper airway topicalization for the use of flexible optic equipment, video laryngoscopy, or combined techniques is essential for success and requires thorough knowledge of the pharmacology of local anesthetics and adjuncts [29]. Awake techniques also present an excellent opportunity to learn and execute proper human factors applications, such as patient guidance and team competencies, enabling safe awake airway management.

#### Emergency front-of-neck access

This rare life-saving procedure requires proper tailored training, as the technique varies between children under 8 years of age and adults [30,31^■^].

#### Tubeless surgery

These oxygenation techniques, such as high-flow nasal oxygen or supraglottic jet ventilation, can be trained on a low-fidelity simulator to learn the application of the necessary equipment and monitoring options.

### Teaching and training modalities

#### Knowledge acquisition

As traditional didactic lectures result in very low retention of knowledge, interactive methods of classroom or individual self-learning are highly recommended. These include self-directed reading, case discussions, video, and gamified problem solving before classroom teaching, short mental visualization before approaching a patient [32], which all can be summarized under the flipped classroom concept.

#### Simulation-based training

This is the [33^■^,^34^] domain of task trainers to learn the use of the equipment and accelerate acquisition of essential dexterity to a nearly self-acting level [35^■^] (e.g. for laryngoscopy, flexible scope intubation, combined techniques, or surgical airway). Direct supervision and immediate feedback are necessary to prevent incorrect steps and the improper application of equipment.

High-fidelity simulation is ideally suited for training in team dynamics and nontechnical skills. However, when technical procedures are embedded into full-scale simulation scenarios, they can be taught with effectiveness comparable to task trainers, albeit at significantly higher cost [36^■■^,37^■^]. Despite this, regular interprofessional simulations focused on complex and often rare scenarios, such as airway obstruction or massive hemorrhage, can substantially enhance patient safety. These simulations help reduce complications by reinforcing standardized protocols, incorporating brief preoperative team huddles that highlight key procedural steps and potential pitfalls, and conducting concise clinical debriefings to review what occurred, what went well, what could be improved, and how to implement those improvements in future practice [38,39].

Extended reality applications for airway management, including VR [40^■■^], augmented reality (AR) and software trainer for flexible scope intubation [19,41,42^■■^]] are becoming broadly available. Reinforced by gamification [43], this allows for repetitive practice of skills or procedures to achieve mastery of the skill in a safe environment without harm to the patient. However, it remains unknown whether these teaching options are able to translate the trained skills into clinical performance.

#### Cadaver labs and anatomical models

Teaching in these settings enables deep understanding [44,45,46^■^] of the relevant complex anatomy and allows practicing surgical airway techniques very close to reality [47^■^]; however, such training depends on the cadavers/models availability, and may be hampered by conflicting schedules.

#### Procedural skills training

Focus on specific advanced procedures and techniques pertinent to anesthesia for head and neck surgery. Examples may include management of staged extubation [48], hemodynamic management and fluid resuscitation in cases of significant blood loss (blood product administration protocols), and goal-directed fluid therapy for free flap head and neck reconstructive surgery [49^■^]. Other training scenarios may include, for example, management of airway during posttonsillectomy bleeding, with immediate interventions aimed at prevention of exsanguination and aspiration, rapid sequence intubation, the management of possible airway obstruction, teaching proper suction technique, patient repositioning, and potential use of a surgical airway, in a highly condensed situation requiring timely decision making and collaborative team performance.

Other emergency training scenarios might address tumor-related bleeding or bleeding during neck dissection, or large vessel injury during the surgery. The focus of such training would be on rapid advanced airway management, hemostasis, decision-making, and interprofessional communication.

## THE TEACHING OF NONTECHNICAL SKILLS IN ANESTHESIA FOR HEAD AND NECK SURGERY

The interdisciplinary teamwork among anesthesiologists, surgeons, nursing staff, and adjunct disciplines (e.g. intensivists) is essential to assure safe outcomes for head and neck surgical patients. The human factors concept takes a holistic view on how people, technology, and the environment interact to influence performance, well-being, and safety. Nontechnical skills are a specific subset of human factors that focus on interpersonal and cognitive skills and social abilities, which complement technical skills to ensure effective and safe task performance (Table 1).

Nontechnical skills of an anesthesiologist would focus on clear communication with the team, making quick decisions under pressure, and fostering awareness of what’s happening in the operating room, whereas human factors would analyze the design of the operating room, the composition of teams, or the hospital’s communication policies.

Several studies highlighted the importance of competent application of nontechnical skills in medicine, especially in high-stakes environments like head and neck surgery. More than 20 years ago, the framework of the anaesthestists’ nontechnical skills (ANTS) [7] was introduced as a validated teaching and assessment tool. The main categories of ANTS are situation awareness, decision making, teamwork, and task management.

Situation awareness, in brief, stands for the ability to recognize, anticipate, and understanding of problems or complications before they arise and the dynamic of the clinical picture (e.g. recognizing early signs of airway compromise or significant blood loss), or in other words: knowing what’s happening around you. An example of a teaching scenario might be efficient time management when dealing with airway obstruction or significant bleeding, to ensure the proper intervention is performed expeditiously and at the right time. Debriefings after such simulated situation awareness scenarios, or after structured observation in the operating room, present educational strategies for improving learning of these nontechnical skills.

Decision making stands for fast, sound judgments under pressure (e.g. managing a ‘can’t intubate, can’t ventilate’ scenario). For teaching purposes, training of closed-loop communication of decisions enhances understanding as it contributes to a shared mental model of all team members. Teaching decision-making can be facilitated in case-based discussions, critical incident analysis, and simulation with escalating complexity. All that promotes reflective practice and encourages explicit verbalization of mental models.

Teamwork (including leadership and communication) relates to interprofessional and interdisciplinary collaboration that emphasizes the effective exchange of information, with a focus on the critical interaction between all allied healthcare professionals in head and neck surgery, aimed at achieving a common goal. Teaching scenarios can include a team time-out with an effective briefing before starting the intervention, aiming to ensure that all team members understand the objectives, risks, and individual responsibilities. That nontechnical skills can be taught during the reflection after team-based simulation scenarios, enhancing the use of structured communication tools (e.g. situation, assessment, background, recommendation) [50], or the debriefing focusing on communication breakdowns, as well as analyzing how to create psychological safety [51] for a speak up culture.

Task management involves prioritization and resource allocation to manage multiple tasks simultaneously during complex procedures. Examples of teaching scenarios may involve complex airway assessment of a (simulated) head or neck surgery patient, airway management planning based not only on the bedside airway assessment tests, but also on preoperative imaging (e.g. computed tomography scan) and nasal endoscopy findings, and discussed with surgeons and patients. Teaching interventions are the training of the use of checklists [17], preoperative briefings (‘huddles’), and simulation to practice efficient workflow and resource utilization.

High-fidelity simulation [52] is the cornerstone for teaching and training nontechnical skills. The realism specific to anesthesia in head and neck surgery comes from the integration of typical difficulties in airway management into the scenarios and the inclusion of particular head and neck complications (e.g. major hemorrhage, airway fire, loss of airway after laryngectomy), which also allows for training both technical proficiency and nontechnical skills in unison.

Learning occurs after the simulation’s action, during the reflection phase in structured debriefing sessions that follow the simulation. Learners are guided by facilitators to reflect on both their technical actions and nontechnical behaviors, receiving constructive and corrective feedback to help the team and individual learners reflect on the procedure, identify what went well, and discuss areas for improvement. This reflection process is crucial for continuous learning and enhancing the quality of performance in clinical practice. Scripted debriefings are particularly helpful for simulation facilitators with limited experience in this area.

In situ simulation with the entire healthcare team within the actual clinical environment serves to train specific emergency procedures or the use of algorithms and protocols to improve team performance [53^■^], but can also identify latent safety threats, organizational flaws, or limitations.

## SPECIAL EDUCATIONAL CONSIDERATIONS FOR THE TEACHING OF AIRWAY MANAGEMENT IN ANESTHESIA FOR HEAD AND NECK SURGERY

Medical education shifted years ago from its traditional focus on time-based or subject-centered apprenticeship, concentrating on knowledge acquisition and a fixed amount of time spent in training, to competency-based medical education (CBME). CBME emphasizes demonstrable mastery of skills and behaviors, which allows learners to progress at their own pace. Such a curriculum is organized around a framework of predefined competences, based on clinical, patient, and society needs. It is highly suitable for an airway rotation in anesthesia for head and neck surgery, as it is flexible, learner-centered, and involves a variety of learning experiences, including direct observation and real-world practice. These competences might be acquired during workshops with hands-on training and simulation (e.g. airway algorithms and failed intubation drill, emergency bleeding management) [54,55]. A CBME curriculum for head and neck anesthesia allows, on the one hand, a modular training with specific modules that integrate both theoretical knowledge and practical skill development, as well as the integration of nontechnical skills. Case-based learning and critical incident debriefing, analyzing real-life or hypothetical critical incidents, seem very suitable to teach nontechnical skills while reflecting the technical skills applied [56]. On the other hand, CBME allows for individualization and tailoring of the learning plan to address the individual needs of each trainee. Such structured clinical training in this specific area of head and neck anesthesia needs to balance structured observation with direct feedback, as well as supervised practice in the operating room, with opportunities for deliberate practice and mastery learning.

The learning outcome can be assessed formative with validated workplace-based assessment tools. Like mini-clinical exams, direct observation of procedural skills, which can evaluate both technical and nontechnical aspects of performance in real clinical settings spaced over time to track a learner’s progress toward achieving competencies. Nontechnical skills can be assessed during observation of behavioral marker systems (e.g. ANTS) in simulated and real clinical environments [57].

## INNOVATIONS AND FUTURE DIRECTIONS

Technological Advancements in Teaching will also enter anesthesia for head and neck, specifically extended reality with options for VR and AR [40^■■^], including artificial-intelligence powered feedback systems to enhance technical skill acquisition [58]. However, its impact on learning and assessment, as well as its cost-effectiveness, is part of ongoing research. The long-term impact of NTS training on patient outcomes and the development of more personalized nontechnical skills training interventions is also unknown.

Escape rooms for learning anesthesia and airway management [59^■^] are slowly entering medical education; however, their value in translating learned competencies into clinical practice is unknown, as well as their cost-effectiveness.

Very little is known about the structures, instructions, and training of airway teachers [33^■^,60^■^], despite the European Union of Medical Specialists issued training requirements for trainers and training institutions in anesthesiology, last in 2022. However, short faculty development interventions are able to improve the instructional competencies of clinical teachers.

Constant concerns are costs and availability of high-fidelity simulators, having interprofessional and interdisciplinary simulation with entire operation room teams and the time away from clinical work of these simulation participants, available dedicated faculty for debriefing in simulation and clinics, supervisors that can ensure that skills and behavior trained in workshops or simulation is environments effectively translate to real-world clinical practice, and sufficient clinical exposure in specialized head and neck cases [61].

At the level of the anesthesia department, strategies need to be developed to ensure the maintenance of high-acuity, low-occurrence competencies relevant to airway management in head and neck anesthesia. Departmental airway leads may improve institutional preparedness for airway management [62], and may facilitate further application and translation of airway management guidelines into clinical practice [63]. However, few anesthesia departments have dedicated programs with a specific, consistent, and evidence-based training curriculum for head and neck anesthesia [5], which takes into account the unique aspects of that field.

## CONCLUSION

The teaching of complex and advanced airway management as part of providing anesthesia for head and neck surgery may use all standard medical education modalities available. The specifics are outlined in the content, including airway management and associated difficulties, as well as the integration of technical and nontechnical skills training in anesthesia for head and neck surgery. Modern head and neck anesthesia education is shifting towards simulation and clinical hands-on training, grounded in CBME that emphasizes both the specific technical competencies in the field and the general nontechnical skills in anesthesia, to enhance patient safety and optimize outcomes.

## Acknowledgements

A.F. and R.G.: conceptualization, methodology, and writing – original draft. V.N.: conceptualization, methodology, and writing - review & editing.

## Financial support and sponsorship

Open access funding was provided by the University of Bern.

## Conflicts of interest

A.F.: AF’s department has received airway equipment, at full, reduced or zero cost, for research and evaluation. A.F. has received no financial gain from this process. V.N.: Director, Stanford Head and Neck Anesthesia and Advanced Airway Management Program, and Director, Stanford Head and Neck Anesthesia Fellowship Program. RG: none related to this article.
